# Airway pressure morphology and respiratory muscle activity during end-inspiratory occlusions in pressure support ventilation

**DOI:** 10.1186/s13054-020-03169-x

**Published:** 2020-07-28

**Authors:** Stella Soundoulounaki, Evangelia Akoumianaki, Eumorfia Kondili, Emmanouil Pediaditis, Georgios Prinianakis, Katerina Vaporidi, Dimitris Georgopoulos

**Affiliations:** 1 Department of Intensive Care Medicine, School of Medicine, University of Crete, Heraklion, Greece; 2 Department of Intensive Care Medicine, University Hospital of Heraklion, Heraklion, Crete Greece

**Keywords:** Esophageal pressure, Gastric pressure, Driving pressure, Protective ventilation

## Abstract

**Background:**

The driving pressure of the respiratory system is a valuable indicator of global lung stress during passive mechanical ventilation. Monitoring lung stress in assisted ventilation is indispensable, but achieving passive conditions in spontaneously breathing patients to measure driving pressure is challenging. The accuracy of the morphology of airway pressure (Paw) during end-inspiratory occlusion to assure passive conditions during pressure support ventilation has not been examined.

**Methods:**

Retrospective analysis of end-inspiratory occlusions obtained from critically ill patients during pressure support ventilation. Flow, airway, esophageal, gastric, and transdiaphragmatic pressures were analyzed. The rise of gastric pressure during occlusion with a constant/decreasing transdiaphragmatic pressure was used to identify and quantify the expiratory muscle activity. The Paw during occlusion was classified in three patterns, based on the differences at three pre-defined points after occlusion (0.3, 1, and 2 s): a “passive-like” decrease followed by plateau, a pattern with “clear plateau,” and an “irregular rise” pattern, which included all cases of late or continuous increase, with or without plateau.

**Results:**

Data from 40 patients and 227 occlusions were analyzed. Expiratory muscle activity during occlusion was identified in 79% of occlusions, and at all levels of assist. After classifying occlusions according to Paw pattern, expiratory muscle activity was identified in 52%, 67%, and 100% of cases of Paw of passive-like, clear plateau, or irregular rise pattern, respectively. The driving pressure was evaluated in the 133 occlusions having a passive-like or clear plateau pattern in Paw. An increase in gastric pressure was present in 46%, 62%, and 64% of cases at 0.3, 1, and 2 s, respectively, and it was greater than 2 cmH_2_O, in 10%, 20%, and 15% of cases at 0.3, 1, and 2 s, respectively.

**Conclusions:**

The pattern of Paw during an end-inspiratory occlusion in pressure support cannot assure the absence of expiratory muscle activity and accurate measurement of driving pressure. Yet, because driving pressure can only be overestimated due to expiratory muscle contraction, in everyday practice, a low driving pressure indicates an absence of global lung over-stretch. A measurement of high driving pressure should prompt further diagnostic workup, such as a measurement of esophageal pressure.

## Background

The driving pressure of the respiratory system is defined as the difference in alveolar pressure between end-inspiration and end-expiration in the absence of muscle activity [1]. During passive mechanical ventilation, the driving pressure of the respiratory system is easily measured, and when chest wall compliance is normal, it is a valid surrogate of lung stretch, the magnitude of which is important for lung injury [2–4]. Indeed, several studies have shown an association between high driving pressure and morbidity and mortality in critically ill and post-operative patients [5–7].

Monitoring lung stretch using driving pressure would be useful also during assisted ventilation [8–11]. However, achieving passive conditions to measure driving pressure in spontaneously breathing patients is challenging. During an end-inspiratory occlusion, the plateau airway pressure (Paw) reflects elastic recoil pressure provided that inspiratory muscle activity has ceased, that the next inspiratory effort has not started, and that there is no expiratory muscle activity. It has been shown that such conditions are present for a brief period (0.25–0.3 s) after the end of neural inspiration [12]. When plateau Paw is obtained by manual end-inspiratory occlusion as suggested [9, 13] during pressure support ventilation, the dissociation between the end of mechanical and neural inspiration, which is frequently observed in this mode, renders the activity of respiratory muscles (inspiratory or expiratory) during occlusion unpredictable. Theoretically, even in the presence of a clear plateau in Paw during occlusion, expiratory muscle activity may be present [14]. Whether it is feasible to confirm the absence of muscle activity by visual inspection of the Paw waveform during a manual end-inspiratory occlusion in pressure support mode has not been studied.

Acknowledging the importance of bedside estimation of lung stretch during pressure support ventilation, we sought to investigate the patterns of responses of inspiratory and expiratory muscles to end-inspiratory occlusions in critically ill patients ventilated in this mode. We examined whether the absence of both inspiratory and expiratory muscle activity can be accurately identified by analyzing the Paw waveform during the end-inspiratory occlusion. To this end, we analyzed the morphology of Paw during end-inspiratory occlusions while concomitantly examining esophageal, gastric, and transdiaphragmatic pressures.

## Methods

This is a retrospective analysis of data obtained from a previously published study [15], and for diagnostic purposes (mainly to titrate the level of assist, and assure protective ventilation in acute respiratory distress syndrome, ARDS), during a 3-year period (2016–2019), in a mixed adult intensive care unit (ICU) of a university hospital. Approval for the anonymous use of the data was obtained from the Hospital’s Ethics Committee.

All patients were ventilated in pressure support mode, and esophageal and gastric balloons were in place. Proper balloon position was confirmed as previously described [15, 16]. Flow (V′), airway (Paw), esophageal (Pes), gastric (Pga), and transdiaphragmatic (Pdi) pressures were measured as previously described [16]. All signals were sampled at 150–200 Hz and analyzed offline.

Patients included in our previous study [15] were ventilated with Servo-i® (Maquet Critical Care, Solna, Sweden). All other patients were ventilated with Evita-XL (Drager, Germany). Because in many patients, the purpose of the measurement was to facilitate assist titration, inspiratory occlusions at 2–4 levels of assist were available for analysis.

### Data analysis

**Fig. 1:**
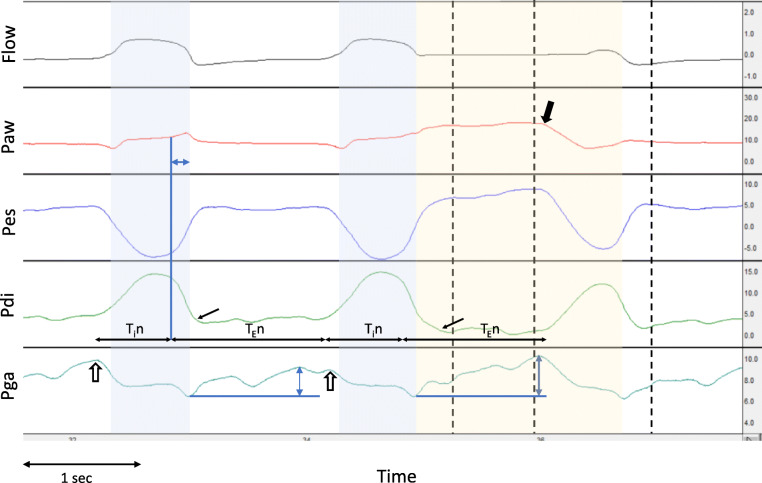
Waveform analysis of end-inspiratory occlusion. Representative waveforms of flow (in l s), airway (Paw), esophageal (Pes), transdiaphragmatic (Pdi), and gastric (Pga) pressures (in cmH_2_O), from one patient, with an end-inspiratory occlusion on the second breath. The blue-shaded area indicates the mechanical inspiratory time, and the yellow-shaded area indicates the end-inspiratory occlusion. Horizontal black arrows indicate the start and end of neural inspiration (T_i_n) and expiration (T_E_n). The point of rapid decline of Pdi (end of neural inspiration) is indicated by the blue vertical line, and the horizontal blue double-headed arrow in Paw indicates the cycling off delay. The small black arrows in Pdi indicate the point of complete relaxation of the diaphragm. Black vertical dashed lines indicate the points at 0.3, 1, and 2 s post-occlusion. The thick black arrow in Paw shows the end of the plateau by the sudden decrease in Paw, due to diaphragmatic contraction during occlusion. Observe that the neural inspiratory and expiratory times are similar in the un-occluded and occluded breath, and the occlusion time is less than 2 s. Expiratory muscle activity is indicated by the rise of Pga, during expiration in the un-occluded breath, and during the end-inspiratory occlusion (blue double-headed arrows show the maximum change). Notice also the decrease of Pga at the onset of inspiration, suggesting the relaxation of expiratory muscles at this point (open arrows)

For each end-inspiratory occlusion, we identified the following time points (Fig. 1): (1) the start of neural inspiration of the occluded and of the preceding breath, as the point of rise of Pdi; (2) the end of neural inspiration as the point of rapid decline of Pdi; (3) the point of occlusion (Paw_occ_), as the point of zero-flow; (4) the point of relaxation of inspiratory muscles, as the point of return of Pdi to baseline; (5) points 0.3 s (Paw_0.3s_), 1 s (Paw_1s_), and 2 s (Paw_2s_) after the occlusion; (6) the end of the plateau in Paw, as the point of decrease in Paw due to inspiratory muscle contraction or release of occlusion with the appearance of expiratory flow (whichever occurred first); and (7) the point of rise of Pga from its value after occlusion, whenever present (Fig. 1). At these time points, we measured Paw, Pes, Pga, and Pdi. The presence of inspiratory effort during the occlusion was identified by an abrupt increase of Pdi. Cycling off delay was quantified as the time difference between the end of neural and of mechanical inspiration (Fig. 1). The presence of expiratory muscle activity during the preceding, un-occluded breath was indicated by (a) a rise of Pga during mechanical expiration with unchanged Pdi and (b) a rapid decrease of Pga at the onset of inspiration of the occluded breath (Fig. 1). The presence of expiratory muscle activity during the end-inspiratory occlusion was indicated by a rise of Pga after the occlusion with a decreasing or constant Pdi. The change in Pga after occlusion was used to quantify expiratory muscle activity (Fig. 1). In some patients, the contraction of expiratory muscles started during mechanical inflation, as indicated by a rapid increase in Pga during the mechanical inflation with Pdi maintained close to end-expiratory values or decreasing. In these cases, accurate quantification of expiratory muscle activity is not feasible since the increase in Pga during mechanical inflation depends both on expiratory muscle contraction and the relationship between lung volume increase and abdominal compliance. In these cases, we used only the change in Pga after occlusion to quantify expiratory muscle pressure, acknowledging the underestimation, to an unknown extent, of expiratory muscle contraction.

**Fig. 2:**
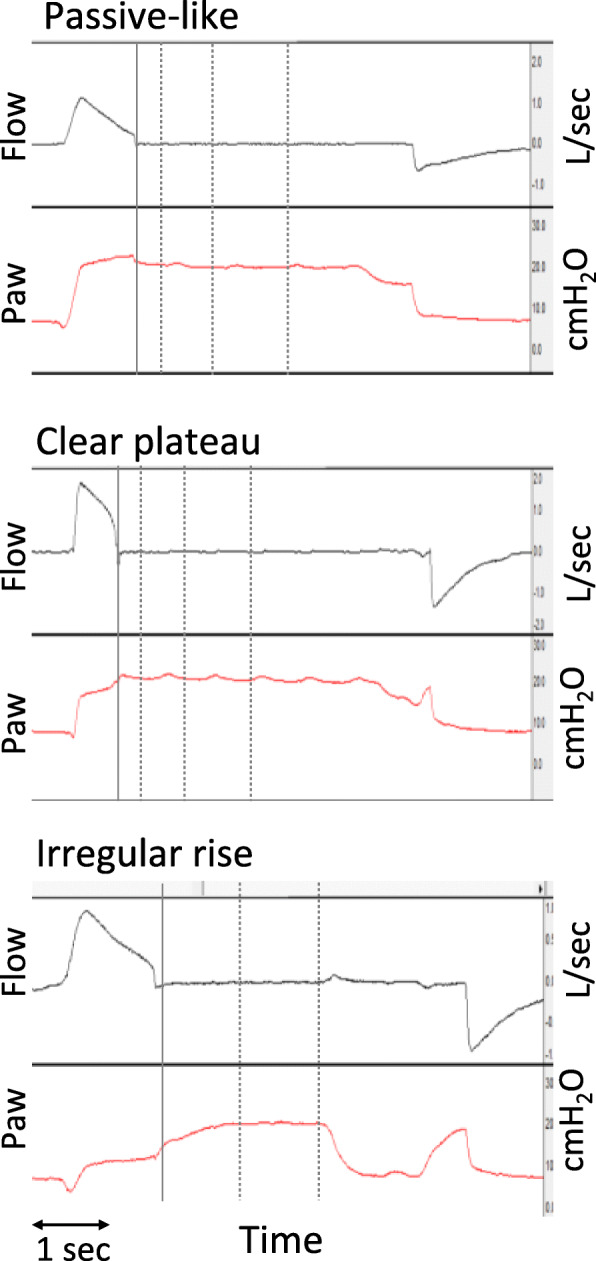
Classification of airway pressure waveform pattern during occlusion. Airway flow (in l/s) and airway pressure (Paw, in cmH_2_O) waveforms representative of the three patterns of Paw during occlusion, from three different patients. The solid vertical line indicates the point of occlusion and subsequent dotted lines the points at 0.3, 1, and 2 s post-occlusion. Each pattern was characterized by the relationships among Paw at different time points relative to the occlusion: occlusion, 0 s (Paw_occ_), 0.3 s (Paw_0.3s_), 1 s (Paw_1s_), and 2 s (Paw_2s_). Upper panel: a “passive-like” pattern with a rapid decrease in Paw (Paw_occ_ > Paw_0.3s_), followed by plateau (Paw_1s_ − Paw_0.3s_ < 1 and Paw_2s_ − Paw_1s_ < 1 cmH_2_O). Middle panel: a “clear plateau” pattern with an early increase in Paw (Paw_occ_ < Paw_0.3s_), followed by plateau (Paw_1s_ − Paw_0.3s_ < 1 and Paw_2s_ − Paw_1s_ < 1 cmH_2_O). Lower panel: an “irregular rise” pattern with a slow increase in Paw (Paw_1s_ − Paw_0.3s_ > 1 cmH_2_O) with plateau (Paw_2s_ − Paw_1s_ < 1 cmH_2_O)

The morphology of Paw after occlusion was classified in three main patterns based on the differences in Paw at the specific time points analyzed (Paw_occ_, Paw_0.3s_, Paw_1s_, and Paw_2s_). In this analysis, a threshold of 1 cmH_2_O was used to characterize the Paw between two consecutive time points as stable, qualifying for “plateau,” or increasing. These patterns as shown in Fig. 2 are: a “passive-like” pattern, characterized by an initial decrease (Paw_0.3s_ < Paw_occ_) followed by plateau (Paw_1s_ − Paw_0.3s_ < 1 and Paw_2s_ − Paw_1s_ < 1 cmH_2_O), similarly to a passive occlusion; a “clear plateau” pattern characterized by a flat or early increase (Paw_0.3s_ ≥ Paw_occ_), followed by plateau (Paw_1s_ − Paw_0.3s_ < 1 and Paw_2s_ − Paw_1s_ < 1 cmH_2_O); and an “irregular rise” consisting of all other patterns of late or continuous increase, with or without plateau (Paw_1s_ − Paw_0.3s_ ≥ 1, or Paw_2s_ − Paw_1s_ ≥ 1 cmH_2_O). The morphology of Pga rise was characterized as continuous rise or constant based on visual inspection of the waveform.

### Statistical analysis

Continuous variables are reported as means and standard deviation (SD) for normally distributed data and as medians and interquartile ranges (IQR) for non-normally distributed data. Categorical variables are presented as percentages. Between-group differences in categorical and continuous variables were compared using the chi-square or Kruskal-Wallis test, respectively. A *p* value of < 0.05 was considered significant. We used IBM SPSS Statistics for Windows version 25 (Armonk, NY) for analysis.

## Results

### Patients and occluded breaths characteristics

The analysis included data from 40 patients (18 from a previous study [15] and 22 from the records of the ICU). Patient characteristics are presented in Table 1. Most patients (90%) had ARDS and had been on mechanical ventilation for a median of 11 days before the measurements.

**Table 1:** Patient characteristics

Patient characteristics, *N* = 40	
Male, *N* (%)	25 (62.5)
Age (mean ± SD)	68 ± 13
APACHE II (mean ± SD)	19 ± 7
Admission diagnosis, *N* (%)	
Acute respiratory failure	16 (40)
Septic shock	10 (25)
Multiple trauma	8 (20)
Post cardiac arrest	2 (5)
Others	4 (10)
Respiratory characteristics and ventilator settings	
ARDS, *N* (%)	36 (90)
Days on MV before study, median (IQR)	11 (7–17)
PEEP cmH_2_O, mean ± SD	7 ± 2
PO_2_/FiO_2_ mean ± SD	235 ± 62
Pressure support, cmH_2_O, mean ± SD	8 ± 3
Tidal volume, L, mean ± SD	0.48 ± 0.08
Respiratory rate, br/min, mean ± SD	23 ± 7

*APACHE II* Acute Physiology and Chronic Health Evaluation II score, *ARDS* acute respiratory distress syndrome, *MV* mechanical ventilation, *PEEP* positive end-expiratory pressure, *PO*_*2*_*/FiO*_*2*_ partial pressure of arterial oxygen to inspired oxygen fraction ratio, *SD* standard deviation, *IQR* interquartile range

In most patients, two to three occlusions were available for analysis. In 19 patients (from ICU records), occlusions were performed at different levels of assist (2–4 levels). Each patient at each level of assist was considered as an individual condition. Eighty-six different conditions (patient/assist level) were identified, and a total of 227 occlusions were available for analysis. Because, in 26 of the 86 conditions, the expiratory muscle activity and/or pattern of Paw during occlusion were different among the occlusions, an analysis per occlusion is presented.

### Analysis of inspiratory muscle activity during occlusion

The relaxation of inspiratory muscles occurred after a median of 0.2 s after occlusion (5–95% range *R*_5–95_ = 0–0.5 s). The median cycling off delay was 0.18 s (*R*_5–95_ = 0.04–0.8 s). The next inspiratory effort appeared during occlusion with a 0.26 s delay (*R*_5–95_ = − 0.04–1.2 s) relative to the patient’s neural timing of the breath, and the median duration of uninterrupted occlusion (“plateau” time) was 2 s (*R*_5–95_ = 1–3.8 s). Only in 16% of occlusions a plateau of 3 s could be maintained uninterrupted, and the respiratory rate in these cases was 16 ± 3 br/min.

### Analysis of expiratory muscle contraction during occlusion

**Fig. 3:**
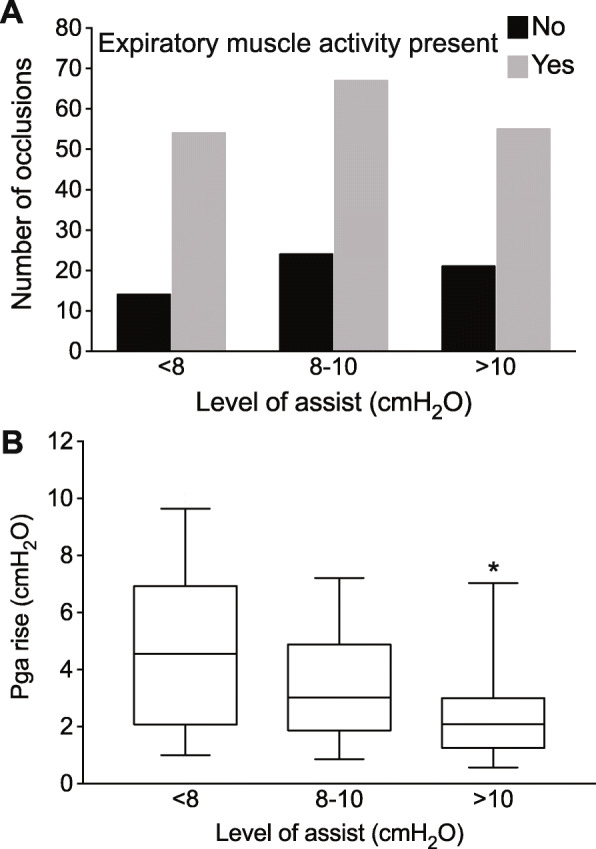
Expiratory muscle activity at different levels of assist. **a** Number of occlusions with expiratory muscle activity present or not, at three different levels of assist. **b** Magnitude of expiratory muscle activity, as indicated by the rise of gastric pressure (Pga) after occlusion, at three levels of assist (only in cases with expiratory muscle activity). Box: interquartile range, whiskers: 5–95 range, line at median, *p < 0.05 for assist level > 10 vs 8–10 cmH_2_O, *p* < 0.0001 for assist level > 10 vs < 8 cmH_2_O

**Fig. 4:**
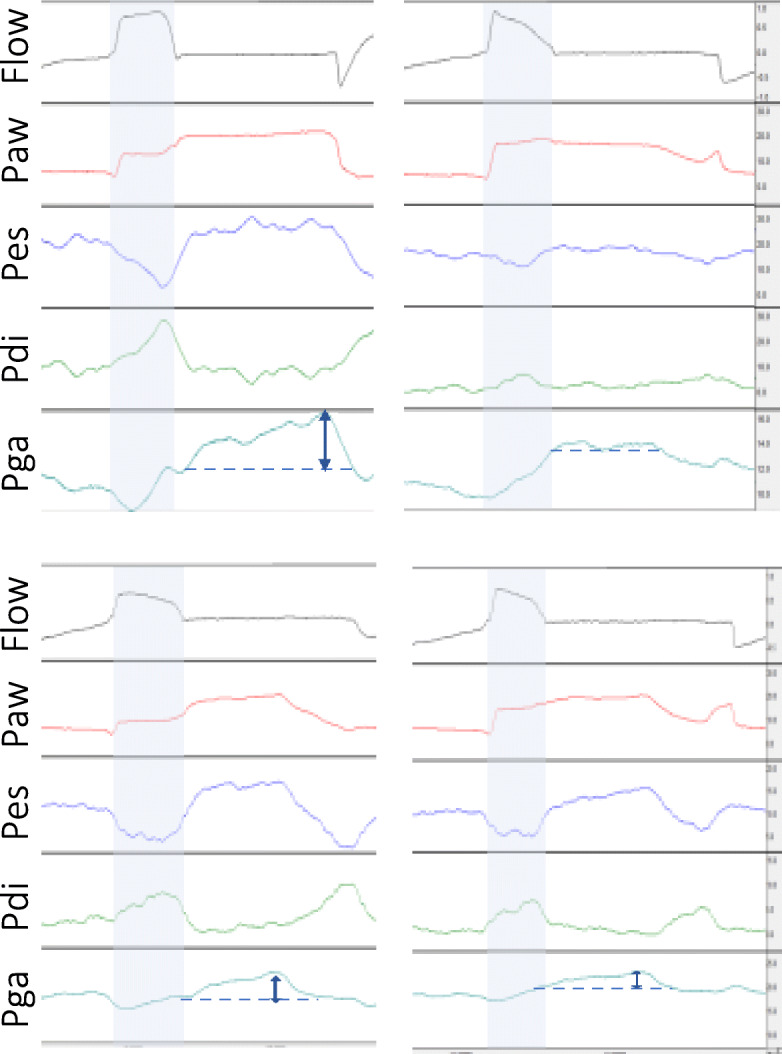
Changes in respiratory muscle activity to increase in pressure support. Representative waveforms of flow (in l/s), airway (Paw), esophageal (Pes), transdiaphragmatic (Pdi), and gastric (Pga) pressures (in cmH_2_O), during an end-inspiratory occlusion, from two patients (upper and lower panel), ventilated with low (left) and higher levels of PS (right panel). The blue arrows indicate the estimated magnitude of expiratory muscle activity

Expiratory muscle activity during occlusion was present in 179 out of 227 cases (79%). In 128 out of these 179 cases, expiratory muscle activity was also present in the preceding (un-occluded) breaths (Fig. 1). As shown in Fig. 3a, expiratory muscle contraction was observed at all levels of assist (chi-square = 0.3). The magnitude, timing, and pattern of expiratory muscle contraction, as indicated by the rise in Pga after occlusion, showed large variability (Additional file 1, Table S1 and Figure A1). Among the occlusions with expiratory muscle contraction, the *R*_5–95_ of Pga rise during occlusion was 1–8 cmH_2_O, with a median of 3 cmH_2_O. With increasing assist levels, the magnitude of Pga rise during occlusion significantly decreased (Figs. 3b and 4). Respiratory rate and the rate of increase of Pdi during inspiration (dPdi/dt, an index of respiratory drive) were not different when expiratory muscle contraction during occlusion was present or not (Additional file 1, Figure A2).

### Analysis of Paw morphology during occlusion

According to the classification presented in data analysis, a “passive-like” pattern was identified in 29 cases, a “clear plateau” pattern in 104 cases, and an “irregular rise” pattern in 94 cases. Characteristics of the occluded breaths for each Paw occlusion pattern are presented in Table 2. Expiratory muscle contraction was present in 52%, 67%, and 100% of cases of Paw with a passive-like, clear plateau, and irregular rise pattern, respectively.

**Table 2:** Characteristics of occluded breaths for each pattern of Paw during occlusion

	Paw pattern		
	Passive-like *N* = 29	Clear plateau *N* = 104	Irregular rise *N* = 94
PS < 8 cmH_2_O, *N* (%)	0 (0)	29 (28)	38 (40)
PS 8–10 cmH_2_O, *N* (%)	5 (17)	47 (45)	35 (38)
PS > 10 cmH_2_O, *N* (%)	24 (83)	28 (27)	21 (22)
Expiratory muscle activity present, *N* (%)	15 (52)	70 (67)	94 (100)
Expiratory muscle in activity in previous breath, *N* (%)	7 (24)	39 (37)	83 (88)
Respiratory rate mean ± SD	19 ± 5	21 ± 4	22 ± 6
dPdi/dt, cmH_2_O/s, median (IQR)	7* (3–8)	11 (6–20)	10 (6–20)
Cycling off delay, s, median (IQR)	0.28* (0.18–0.56)	0.18 (0.14–0.28)	0.18 (0.12–0.24)

*Paw* airway pressure, *PS* pressure support, *dPdi/dt* rate of change of transdiaphragmatic pressure, *SD* standard deviation, *IQR* interquartile range

*p < 0.01 for passive-like pattern vs clear plateau and irregular rise pattern

### Measurement of driving pressure

Τhe measurement of driving pressure was assessed at the pre-specified time points after occlusion, at 0.3 s, at 1 s, and at 2 s. A new inspiratory effort interrupted the occlusion, precluding measurement in 47% of cases at 2 s, and in 3% of cases at 1 s. Relaxation of inspiratory muscles had occurred in 80% of cases at 0.3 s, and in 99% of cases at 1 s. An increase in Pga was present in 50%, 71%, and 74% of total cases at 0.3, 1, and 2 s, respectively. Having identified that an irregular rise pattern in Paw is invariably associated with expiratory muscle contraction, a separate analysis was performed, excluding occlusions with an irregular rise pattern. In the remaining 133 cases, in which a plateau could be considered valid, an increase in Pga was present in 46%, 62%, and 64% of cases at 0.3, 1, and 2 s, respectively. The median increase in Pga was 1 cmH_2_O at all time points (*R*_5–95_ = 0–2 cmH_2_O at 0.3 s, *R*_5–95_ = 0–3 cmH_2_O at 1 and 2 s), and it was greater than 2 cmH_2_O in 10% of all cases at 0.3 s, in 20% of cases at 1 s, and in 15% of cases at 2 s.

## Discussion

This study was a retrospective analysis of the morphology of Paw waveform during end-inspiratory occlusions in pressure support ventilation, and its correlation with the presence of inspiratory and expiratory muscle activity. The end-inspiratory occlusions analyzed were obtained from patients in the recovery phase of ARDS, who had been for a median of 11 days on mechanical ventilation. The main finding of this study is that the pattern of Paw during an end-inspiratory occlusion cannot assure the absence of expiratory muscle activity and, thus, the accurate measurement of plateau and driving pressure.

In this study, we chose to evaluate the morphology of Paw not by visual inspection, to avoid inter-observer variability, but by classifying based on the changes at pre-specified time points. To implement this approach, we had to select the thresholds for the classification of Paw patterns. The 0.3 s time point was chosen based on a previous study showing that at up to 0.3 s after the end of neural inspiration there is minimal respiratory muscle activity [12], acknowledging that, in pressure support mode, the end-inspiratory occlusion may not occur at the end of neural inspiration. We chose to study the 1st and 2nd second after occlusion as reasonable, easily identifiable time points at the bedside. Τhe threshold of 1 cmH_2_O to characterize the Paw between two-time points as stable or increasing was chosen because smaller pressure differences can be caused by cardiac oscillations [12] and cannot be accurately measured at the bedside on the ventilator screen. Using these thresholds, we classified the morphology of Paw in the three commonly observed patterns during end-inspiratory occlusions [8]. The “passive-like” pattern was found in patients at a higher level of assist and was associated with lower respiratory drive, as indicated by dPdi/dt. Even in these conditions, expiratory muscle activity was present in half of the cases, without clear distortion of the “plateau” in Paw waveform. A “clear plateau” was the most commonly observed pattern. Importantly, expiratory muscle activity was present in two-thirds of such cases and in all levels of assist. Finally, when a change greater than 1 cmH_2_O was observed between any two sequential time points (0.3–1–2 s) after occlusion, classified as irregular rise pattern, expiratory muscle activity was invariably present.

During the end-inspiratory occlusions, the presence of inspiratory effort was easily recognized in the vast majority of cases. Inspiratory efforts interrupted the occlusion at a time determined by the patient’s breathing rate and neural inspiratory/expiratory time. These data indicate that during pressure support ventilation, the duration of occlusion cannot be pre-selected to be 3 s, as in passive ventilation, but can be expected to be close to the expiratory time of the previous breath.

We observed that in more than half of the patients (22/40) and 128 out of 227 occlusions, expiratory muscle activity was present in the un-occluded breaths. In all of these cases, expiratory muscle activity was present also during the end-inspiratory occlusions. These findings are not unexpected, as phasic recruitment of expiratory muscles occurs during high ventilatory demands (i.e., during exercise) and whenever a relatively increased load, due to abnormal mechanics or weak muscles, is imposed on the inspiratory muscles [14, 17]. The patients included in this study were in the recovery phase of critical illness, and most had ARDS, and thus, a relatively increased inspiratory muscle load was likely, due to impaired mechanics and/or muscle weakness. Moreover, they had relatively high minute ventilation, of 11 L/min on average. These ventilatory demands, which likely underestimate the brain demands [18], are similar to those observed in light exercise, where phasic expiratory muscle activity normally occurs [17]. Other studies in critically ill patients have also shown that expiratory muscle activity is often present during assisted ventilation [19, 20]. We also observed that in additional 51 cases (15 patients), expiratory muscle activity was present only during end-inspiratory occlusion, a phenomenon likely due to behavioral responses and the infinite resistive load imposed by the occlusion [12, 14, 21]. Therefore, expiratory muscle activity during end-inspiratory occlusion is not an uncommon finding in critically ill patients ventilated in pressure support mode. A clear plateau in Paw during end-inspiratory occlusion does not exclude expiratory muscle activity. This finding is in line with a previous study [14] showing that, during end-expiratory occlusions, the pattern of expiratory muscle contraction may result in a sustained increase in Paw and clear plateau, rendering the measurement of end-expiratory elastic recoil pressure (PEEPi) unreliable. Thus, similar to an end-expiratory occlusion [14], expiratory muscle contraction may also result in plateau in Paw during an end-inspiratory occlusion, and thus, in overestimation of end-inspiratory alveolar pressure.

The presence of expiratory muscle contraction during end-inspiratory occlusions has been studied in proportional assist ventilation [12], a mode in which the end of mechanical and neural inspiration is tightly linked. This study [12] showed that the occurrence of events, such as expiratory muscle contraction, progressively increased over time after the occlusion, starting at 0.3 s post-occlusion, with an incidence of 10%. During pressure support ventilation, the end of mechanical and neural inspiration is not tightly linked, and a median cycling off delay of 0.18 s (*R*_5–95_ = 0.04–0.8 s) was observed in this study, which may explain the presence of expiratory muscle contraction even immediately after occlusion. The presence of expiratory muscle contraction even before the termination of mechanical inflation in critically ill patients ventilated in pressure support mode has been previously reported [20]. Therefore, the presence of conditions enabling accurate measurements of driving pressure in pressure support was not supported by the findings of this study. No pattern of Paw excluded the presence of expiratory muscle activity, and no time point was identified before the appearance of expiratory muscle activity and after the relaxation of inspiratory muscles. The extent of overestimation of driving pressure could not be accurately computed, but in 20% of cases, an overestimation of at least 2 cmH_2_O was observed.

This study design does not provide information on the prevalence of expiratory muscle activity in patients during assisted ventilation, which will likely vary, depending on the patient population examined and the time during the course of weaning. The correlation between inspiratory muscle strength, inspiratory load (respiratory system mechanics), and presence of expiratory muscle activity was not examined. The expiratory muscle activity was assessed using Pga and Pdi, but without the use of electromyography, which would provide more accurate information on the timing of expiratory muscle contractions. Therefore, the magnitude of Pga rise was underestimated in some cases. Although the pattern of Paw could not assure the absence of expiratory muscle activity, other means to assess it, such as physical examination, ultrasound, or electromyography, were not examined in this study.

The findings of this study have some important clinical implications. Firstly, simple rules were identified to predict the duration of the inspiratory hold (slightly longer than the observed expiratory time) and to identify invalid end-inspiratory occlusions due to expiratory muscle activity (increase greater than 1 cmH_2_O between two consecutive time points). Additionally, it was confirmed that the driving pressure measured during the inspiratory hold may only be overestimated, because inspiratory efforts are easily recognized, suggesting that a low driving pressure can reliably exclude global lung over-distention. On the other hand, the finding of a high driving pressure, which has been associated with adverse patient outcome [9], requires further investigation. A high driving pressure could result from expiratory muscle activity, increased inspiratory muscle effort, impaired lung mechanics, and/or decreased chest wall compliance. Because all of these conditions are associated with adverse outcomes, but targeted management is required, it seems prudent to implement further diagnostic workup, such as measurement of esophageal pressure, to estimate respiratory effort, drive, and transpulmonary pressures.

## Conclusions

The results of this retrospective analysis of Paw morphology during end-inspiratory occlusion in pressure support in critically ill patients indicate that the pattern of Paw may confirm the presence of both inspiratory and expiratory muscle activity, but not the absence of expiratory muscle activity. These data show that the passive conditions required for accurate measurement of driving pressure in an end-inspiratory occlusion cannot be assured using just Paw morphology. Yet, because driving pressure can only be overestimated due to expiratory muscle activity, in everyday practice, a low driving pressure indicates the absence of global lung over-stretch. A measurement of high driving pressure should prompt further diagnostic workup, such as measurement of esophageal pressure.

## Supplementary information

**Additional file 1: Table S1.** Timing of expiratory muscle contraction during end-inspiratory occlusion. **Figure A1.** Patterns of expiratory muscle activity during end-inspiratory occlusion. **Figure A2.** Respiratory rate and drive in the presence or absence of expiratory muscle activity during occlusion.

## Data Availability

The datasets used in the current study are available from the corresponding author on reasonable request.

## Abbreviations

ARDS: Acute respiratory distress syndrome
ICU: Intensive care unit
Paw: Airway pressure
Paw_occ_: Airway pressure at occlusion
Paw_0.3_: Airway pressure at 0.3 s after occlusion
Paw_1_: Airway pressure at 1 s after occlusion
Paw_2_: Airway pressure at 2 s after occlusion
Pes: Esophageal pressure
Pga: Gastric pressure
Pdi: Transdiaphragmatic pressure
dPdi/dt: Rate of change of Pdi during inspiration
V′: Flow
SD: Standard deviation
IQR: Interquartile range
*R*_5–95_: Range between the 5th and 95th percentile
