# Supplementary material for: Airway pressure morphology and respiratory muscle activity during end-inspiratory occlusions in pressure support ventilation
Source: Crit Care. 2020 Jul 28;24:467. doi: 10.1186/s13054-020-03169-x (PMC7385937; doi:10.1186/s13054-020-03169-x)
Supplement: Supplementary file 1 — Additional file 1: Table S1. Timing of expiratory muscle contraction during end-inspiratory occlusion. Figure A1. Patterns of expiratory muscle activity during end-inspiratory occlusion. Figure A2. Respiratory rate and drive in the presence or absence of expiratory muscle activity during occlusion. [file 13054_2020_3169_MOESM1_ESM.docx]

Airway pressure morphology and respiratory muscle activity during end-inspiratory occlusions in pressure support ventilation.

Additional File 1

**Table S1: Timing of expiratory muscle contraction during end-inspiratory occlusion**

| Timing of appearance of expiratory muscle contraction | |
| --- | --- |
| From the end of neural inspiration  (median, 5-95% range) | 0.40, 0.08 -1.08 sec |
| From the point of occlusion (median, 5-95% range) | 0.19, 0 - 1 sec |
| Expiratory muscle activity present at the time of occlusion (% of patients) | 25 |

**Figure A1: Patterns of expiratory muscle activity during end-inspiratory occlusion**

**
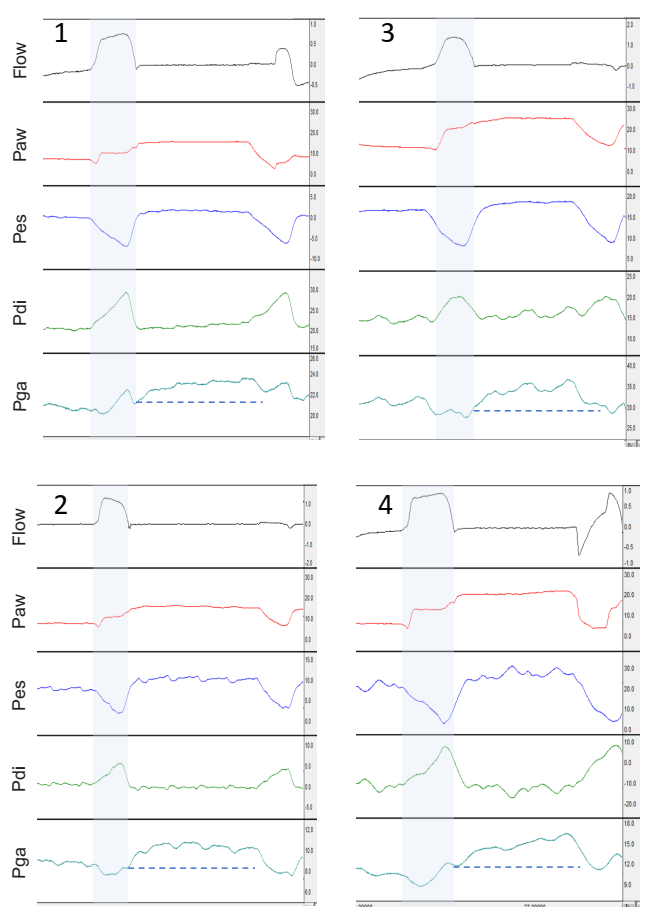
**

Representative waveforms of flow (l/sec), airway (Paw), esophageal (Pes), transdiaphragmatic (Pdi), and gastric (Pga) pressures (cmH_2_O), during an end-inspiratory occlusion, from four patients (1-4). The blue shaded area indicates the mechanical inspiratory time and the dashed blue lines show Pga at the beginning of occlusion (zero flow). The expiratory muscle activity in patients 1 and 2 is rather constant during the occlusion (tonic), and this was observed in 47% of cases. The expiratory muscle activity in patients 3 and 4 progressively increases in patients, and this was observed in 53% of cases. In all cases a plateau can be identified in Paw.

**Figure A2: Respiratory rate and drive in the presence or absence of expiratory muscle activity during occlusion**


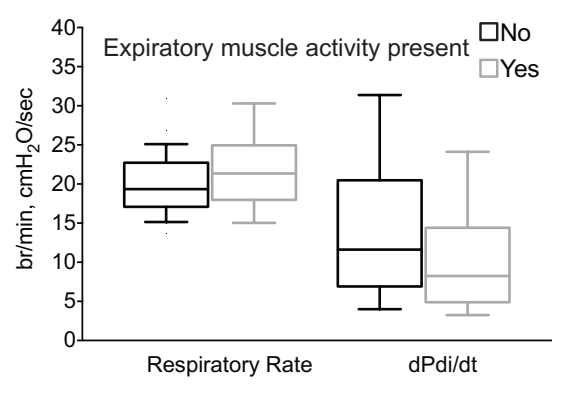


Respiratory rate in breaths per minute (br/min) and respiratory drive as indicated by the rate of change of transdiaphragmatic pressure during inspiration (dPdi/dt) of the occluded breath, in the presence or absence of expiratory muscle activity during the end-inspiratory occlusion. Box: interquartile range, whiskers: 5-95 range, line at median
